# Supplementary material for: What Works to Improve Nutrition and Food Sustainability across the First 2000 Days of Life: A Rapid Review
Source: Nutrients. 2022 Feb 9;14(4):731. doi: 10.3390/nu14040731 (PMC8878998; doi:10.3390/nu14040731)
Supplement: Supplementary file 1 [file nutrients-14-00731-s001.zip › nutrients-1541971-supplementary.pdf]

Table S1. Search terms

Category	Search String
Population	pregn* OR p#ediatr* OR infan* OR toddler* OR preschool* OR "pre school*" OR "pre-school*" OR baby OR babies OR neonat*
Intervention	diet* OR nutrit* OR "family meal*" OR "responsive feeding" OR discretionary OR breastfeeding OR "breast feeding" OR "bottle feeding" OR bottlefeeding OR "formula feed*"
	fruit OR vegetable* OR "soft drink" OR "sugar sweetened beverage"
	food N2 supply OR sustainab* OR culture OR environment OR practice* OR afford* OR access* OR practices OR literacy
	"community market*" OR "farmers market*" OR "local government" OR "local council" OR "local authorit*" OR "health service*" OR "health setting*" OR "child care" OR childcare
	"point of purchase" OR "marketing" OR "outdoor advertising" OR "policy"
Study types	"systematic review" OR "meta analys*"
